# Development and Validation of an Extended Adult Vaccine Hesitancy Scale in Greek-Speaking Populations

**DOI:** 10.3390/vaccines14070628

**Published:** 2026-07-17

**Authors:** Andria Hadjikou, Irene Heraclidou, Alexandros Heraclides

**Affiliations:** 1 Department of Health Sciences, School of Life and Health Sciences, European University Cyprus, 6 Diogenis Street, 2404 Engomi, Cyprus; 2 Department of Psychology, Faculty of Social Sciences and Education, University of Cyprus, 1678 Nicosia, Cyprus

**Keywords:** vaccine hesitancy, adult Vaccine Hesitancy Scale, VHS, validation, validity, reliability

## Abstract

Background: Vaccine hesitancy (VH) is a complex global public health threat requiring validated tools for its assessment globally. This study aimed to evaluate an extended adult Vaccine Hesitancy Scale (aVHS) among Greek-speaking adults. Methods: This study evaluated a newly developed extended version of a widely used aVHS, incorporating five additional items on long-term safety, risk–benefit evaluation, vaccine-related harm, scientific credibility, and perceived alternatives, on a cross-sectional sample of 491 adults in Greece and Cyprus. Cross-cultural adaptation of the extended aVHS involved translation, back-translation, and pilot testing. Structural validity was assessed using parallel analysis, exploratory factor analysis (EFA), and reliability using Cronbach’s α and McDonald’s ω. Criterion validity was assessed against focus-group classification using ROC analysis in 68 participants. Results: A one-factor EFA solution appeared the most parsimonious, showing strong loadings for the original and extended aVHS (0.67–0.86 and 0.65–0.87), with similar explained variance (60.31% and 60.06%). The one-factor solution was confirmed by parallel analysis for both scales. Internal consistency was excellent, and slightly higher for the extended than the original aVHS (α = 0.95; ω = 0.95 vs. α = 0.92; ω = 0.93). The five newly added items performed strongly, with item–rest correlations of 0.69–0.85 and loadings of 0.72–0.87. Criterion validity was excellent, with a slightly higher AUC (0.991 vs. 0.981) and numerically higher classification performance for the extended than the original aVHS. Conclusions: The extended aVHS is a psychometrically coherent tool that improves the performance of the original scale among Greek-speaking individuals. This scale may enhance VH surveillance, aiding targeted vaccination communication strategies.

## 1. Introduction

Vaccination is the most effective public health intervention for preventing infectious diseases, reducing morbidity and mortality, and protecting individuals and communities [1]. However, the success of vaccination programs depends not only on vaccine availability but also on public acceptance. This is increasingly important as infectious disease outbreaks have become more frequent and diverse, while global travel, urbanization, land-use changes, and environmental pressures continue to intensify pandemic risks [2,3]. Recent events, including the 2026 multi-country hantavirus cluster linked to cruise ship travel, show how infectious threats can cross borders and require coordinated public health responses, even without widespread community transmission [4]. The COVID-19 pandemic further demonstrated that vaccination can become a source of uncertainty and public debate when safety concerns, low trust in authorities, perceived disease risk, and misinformation coexist [5,6].

According to the WHO Strategic Advisory Group of Experts (SAGE) Working Group, vaccine hesitancy (VH) is defined as the delay in acceptance or refusal of vaccination despite the availability of vaccination services. It is a complex and context-specific phenomenon that varies across time, place, and vaccine type [7,8]. Within the SAGE framework, VH is shaped by confidence, complacency, and convenience [7,9]. Confidence reflects trust in vaccine safety and effectiveness, health services, and policymakers; complacency refers to low perceived risk from vaccine-preventable diseases; and convenience concerns whether vaccines are accessible, affordable, and available. Broader social and cultural factors, including community norms, misinformation, and institutional trust, remain critical determinants of vaccine acceptance [10].

Because VH is an attitudinal construct, it cannot be fully captured by vaccination status alone. Reliable and valid measurement tools are therefore needed to assess it more comprehensively, identify hesitant population groups, and guide targeted public health strategies [10,11]. The Vaccine Hesitancy Scale (VHS) is a widely used and psychometrically evaluated instrument initially developed for childhood vaccination and later adapted for adults to assess general vaccination attitudes (adult Vaccine Hesitancy Scale—aVHS) [8,11,12]. The aVHS is especially important because adult vaccination decisions involve routine vaccines, booster doses, high-risk groups, and responses to future infectious threats [13,14]. Although adult-adapted versions support the relevance of this framework, further validation and refinement remain necessary across countries, languages, and population settings, as demonstrated by recent validation efforts in various European countries [13,15,16].

The Greek version of the aVHS was validated in a community-dwelling Greek population and was shown to be reliable and valid for assessing vaccine-related attitudes in the Greek society [16]. However, the original validation evaluated the existing 10-item adult scale and did not extend the instrument to address the limited coverage of risk-perception items, as previously recommended in validation work on the parental and adult versions of the scale [11,12,16]. Therefore, further development and validation of an extended adult scale is warranted to capture the complexity of modern vaccine-related concerns.

In this context, the present study developed, to our knowledge, the first extended aVHS by adding new risk- and safety-related items to capture risk perception, safety confidence, risk–benefit evaluation, long-term safety concerns, and perceived alternatives to vaccination more comprehensively. The resulting scale was psychometrically evaluated among adults in Greece and Cyprus, two culturally related Eastern Mediterranean countries with comparable health system contexts. We examined the cross-cultural adaptation, reliability, and validity of the extended aVHS and compared its performance with the original 10-item aVHS.

## 2. Materials and Methods

### 2.1. Study Design, Setting, and Participants

A cross-sectional study was conducted in Greece and Cyprus between January 2022 and April 2023 to investigate the determinants of VH and vaccine refusal among Greek-speaking adults. The broader study, which examined novel determinants of VH, has been described in detail elsewhere [17]. The present analysis focused specifically on the development and psychometric evaluation of an extended version of the aVHS, comparing its performance with the original 10-item aVHS [11,17].

In brief, the study was conducted during and immediately after the COVID-19 pandemic, a period marked by restricted population mobility and limited access to participants. Consequently, random probability sampling was not feasible. Participants were recruited through a broad targeted and proportional quota-sampling approach, reaching a response rate of 52%. Sampling quotas were informed by the adult population structure of Greece [18] and Cyprus [19], using the most recent national census data regarding gender, age group, and geographical region. This strategy aimed to approximate the adult population structure of the two countries and to increase demographic, geographic, and socioeconomic heterogeneity.

Participants were recruited both online and in person. Online recruitment was based on targeted dissemination across different population groups, while in-person recruitment was conducted, where feasible, in high-traffic public locations, including hospitals, supermarkets, shopping areas, and central urban or rural locations. The recruitment strategy involved targeted sampling from various socioeconomic strata (e.g., more affluent and less affluent areas) to enhance geographic and socioeconomic diversity. The study successfully captured participants from all nine geographical regions of Greece (Attica, Central Greece, Central Macedonia, Eastern Macedonia and Thrace, Epirus, Peloponnese, Thessaly, Crete, and the Aegean Islands) and all five districts of the Republic of Cyprus (Nicosia, Limassol, Larnaca, Paphos, and Famagusta). Although the strategy aimed for representativeness, the final sample showed an over-representation of females (64.2%) and a slight under-representation of individuals aged over 60 years (12.6%). The sample size for the present validation (n = 491) exceeded the minimum requirement of 360 participants calculated for the primary study’s power analysis, supporting sufficient statistical power for psychometric evaluation [7,8,17]. Eligible participants were adults aged ≥18 years who had lived in Greece or Cyprus during the previous three months, were able to read Greek, and provided informed consent.

Participants were excluded if they were younger than 18 years, had not lived in Greece or Cyprus during the previous three months, were unable to read Greek, did not provide informed consent, or had incomplete data for the original or extended aVHS scales or for key sociodemographic variables required for the present validation analysis.

The present psychometric validation focused on 491 adults with complete data on the original and extended aVHS scales and on key sociodemographic variables. This sample size was considered adequate for exploratory factor analysis of the 15-item extended aVHS, corresponding to approximately 32.7 participants per item. This exceeds commonly used participant-to-item recommendations for EFA and is well above commonly recommended absolute sample-size thresholds for psychometric scale evaluation.

Data were collected using a structured, self-administered questionnaire created in Microsoft Forms and disseminated by trained research assistants. Before the main data collection, the questionnaire was pilot tested in an initial sample of 69 participants to ensure item intelligibility, comprehension, cultural appropriateness, and questionnaire length, following COSMIN guidelines [20,21]. Pilot testing did not identify any major issues, and the instrument was subsequently used for the main data collection. Sixty-eight of the pilot phase participants also took part in the focus-group evaluation phase.

### 2.2. Focus Groups and Qualitative Data Synthesis

The focus-group evaluation phase (n = 68) was conducted to refine the extended aVHS and establish a qualitative reference standard for criterion validation. Eight focus groups were held, with 8–10 participants per group, following methodological guidelines for psychometric pre-testing and thematic saturation, which typically require at least 15–20 participants [22]. This sample size also exceeded the recommended minimum participant-to-item ratio (4:1) for the qualitative assessment of the newly developed items and the requirements for content validation [23].

Interviews were conducted in person, based on the WHO SAGE 3Cs framework. Participants answered open-ended questions and engaged in facilitated discussion specifically focused on vaccine safety and risk perception, including the following topics:

1. Long-term safety: “What are your main concerns regarding the potential long-term effects of a vaccine on your health years from now?”
2. Scientific and institutional trust: “Do you believe pharmaceutical companies and health authorities disclose all necessary information about side effects, or do you feel some data is being withheld?”
3. Development speed and rigor: “How confident do you feel about the safety of new vaccines that are developed and approved within accelerated timeframes?”
4. Risk–benefit evaluation: “How do you personally weigh the risk of potential vaccine side effects against the risk of becoming seriously ill from the disease itself?”
5. National health system reliability: “To what extent do you trust the national health recommendations provided by the national authorities in your country regarding vaccine safety?”
6. Perceived alternatives: “Some believe it is safer to gain immunity through natural infection rather than vaccination; what is your perspective on this?”

All sessions were audio-recorded and transcribed verbatim. The resulting qualitative data were analyzed using reflexive thematic analysis to identify, analyze, and report patterns of meaning across the datasets. Two independent researchers initially coded a subset of transcripts using a hybrid inductive and deductive approach, anchored in the WHO SAGE 3Cs framework (confidence, complacency, convenience). Discrepancies in coding were resolved through consensus, yielding a finalized codebook applied to all transcripts.

Based on their expressed attitudes during the 60–90 min sessions, participants were qualitatively classified as “not hesitant,” “somewhat hesitant,” or “very hesitant”. This classification was achieved by synthesizing the emerged themes per participant against predefined rubric criteria reflecting the degree of systemic trust and risk-aversion. This classification served as the qualitative reference for validating the scale’s discriminatory ability in identifying vaccine-hesitant individuals.

### 2.3. Quantitative Assessment

#### 2.3.1. Sociodemographic Characteristics

The study questionnaire assessed key sociodemographic and socioeconomic variables, including gender, age, country of residence, area of residence, marital status, educational attainment, and income. Age was categorized as 18–30, 31–40, 41–50, 51–60, or >60 years, and country of residence as Greece or Cyprus. Area of residence was derived from participants’ self-reported city, town, or village and was initially classified using local demographic criteria as urban (more than 10,000 residents), semi-urban (2000–10,000 residents), or rural (fewer than 2000 residents). For the present analysis, area of residence was grouped as urban versus semi-urban/rural due to low sample size in the middle category. Marital status was categorized as unmarried, married/cohabiting, or divorced/separated/widowed, again due to low sample size in the relevant categories. Educational attainment was assessed as the highest qualification acquired and classified as up to high school, college/BSc, or MSc/PhD, while household gross monthly income was grouped as ≤1000, 1001–2000, or >2000 Euro.

#### 2.3.2. The Original Adult Vaccine Hesitancy Scale

The 10-item aVHS used in the present study was based on the adult-adapted VHS, originally developed to assess parental attitudes toward childhood vaccination [11]. Shapiro et al. [11] psychometrically evaluated the parental VHS and supported a two-factor structure after excluding one poorly performing item. Specifically, item 10, concerning the perceived lack of need for vaccines against diseases that are no longer common, was flagged as unreliable because it loaded similarly on both factors and was therefore removed from their final nine-item factor structure. The final structure thus comprised the following:

- The “Lack of Confidence” factor included items on the following topics:
  1. Vaccine importance (item 1);
  2. Effectiveness (item 2);
  3. Community benefit (item 3);
  4. Benefits of government-recommended vaccines (item 4);
  5. Trustworthy vaccine information (item 6);
  6. Self-protection through vaccination (item 7);
  7. Healthcare-provider recommendations (item 8).
- The “Risk Perception” factor included items on the following topics:
  8. Concerns about newer vaccines (item 5);
  9. Serious adverse effects (item 9) [11].

Luyten et al. later adapted the wording to ensure that the 10-item scale was applicable to general vaccination attitudes in adults [13]. Similar adult-adapted cross-cultural validations have been successfully replicated in other European countries to evaluate population-specific vaccination attitudes [13,15,16], while, in a Greek-speaking population, the specific scale resulted in a single-factor structure [16].

This scale utilized a five-point Likert format ranging from “strongly disagree” to “strongly agree.” Before total score calculation, all items were coded so that higher item scores consistently indicated greater VH. Vaccine confidence items were reverse-scored, so that disagreement with positive vaccine statements contributed to higher hesitancy. Vaccine risk items and the item on perceived lack of need for vaccination (item 10) were scored directly, so that stronger agreement contributed to higher hesitancy. Therefore, total possible scores ranged from 10 to 50. Higher scores indicated greater VH, whereas lower scores indicated lower VH (Supplementary Table S1).

#### 2.3.3. Development of the Extended Adult Vaccine Hesitancy Scale

The extended aVHS retained the 10 items of the original adult-adapted VHS and added five theoretically informed items to broaden the assessment of vaccine risk and safety-related beliefs, consistent with recommendations by Shapiro et al. to strengthen risk-perception item coverage [11].

Item selection was guided by the WHO SAGE 3Cs framework, particularly the confidence and complacency domains, and by previous validation work on the parental VHS and its adult-adapted versions, which highlighted the need for broader coverage of vaccine risk perception [7,11]. Accordingly, the added items were selected to capture long-term safety concerns, risk–benefit appraisal, perceived vaccine-related harm, scientific credibility and safety confidence, and perceived alternatives to vaccination.

The five newly added items cover the following domains:

1. Long-term side-effect concerns (item 11);
2. Perceived risk–benefit balance (item 12);
3. Perceived probability of serious vaccine-related harm (item 13);
4. Perceived scientific credibility and safety of vaccination (item 14);
5. Perceived safer or easier alternatives to vaccination (item 15).

Item 15 was designed to capture perceived alternatives to vaccination, including beliefs that non-vaccine approaches may be safer, easier, less burdensome, or otherwise preferable to vaccination. Since perceived safety and perceived ease are related but not identical concepts, this item should be interpreted as reflecting a broader preference for alternatives to vaccination rather than safety alone.

Items 11, 12, and 15 were scored directly toward greater hesitancy, as agreement reflected stronger vaccine risk concerns or preference for alternatives. Items 13 and 14 were reverse-scored, as agreement reflected greater confidence in vaccine safety and scientific credibility. After recoding, all 15 items were summed to produce the total extended aVHS score, with higher scores indicating greater VH (Supplementary Table S2 for English version and Supplementary Table S3 for Greek version).

### 2.4. Cultural Adaptation of the Greek Version of the Extended aVHS

To support linguistic and conceptual equivalence, the newly developed items underwent a rigorous translation and back-translation process. The items were independently translated from English into Greek and then back-translated into English by bilingual experts, with an emphasis on preserving conceptual and contextual meaning rather than literal wording.

During the pilot and focus-group stages, the feasibility, face validity, and comprehensibility of the Greek wording were evaluated through cognitive pre-testing. Specifically, participants were asked to paraphrase each item to verify that their interpretation was consistent with the intended construct and to identify potential ambiguities, cognitive burden, or misunderstanding, particularly for reverse-scored statements and risk-perception items. Based on this feedback, minor linguistic and syntactic refinements were made to improve clarity and ease of comprehension without altering the underlying psychometric meaning of the items. No systematic comprehension difficulties were identified for reverse-scored or risk-perception items. The final extended aVHS Greek version was considered linguistically appropriate and culturally relevant for Greek-speaking adults in Greece and Cyprus.

### 2.5. Statistical Analysis

#### 2.5.1. Demographic Characteristics

For categorical variables, frequencies and percentages were calculated. Continuous variables were reported as means with standard deviation (SD) or medians with interquartile range (IQR), depending on their distribution. Original and extended aVHS scores were compared across participant characteristics using independent-samples *t*-tests and one-way analysis of variance (ANOVA), or non-parametric equivalents where appropriate.

#### 2.5.2. Evaluation of Structural Validity and Internal Consistency

Structural validity of both the standard and extended aVHS was evaluated using exploratory factor analysis (EFA) to determine whether the items measured a common VH dimension [8,13,16]. The suitability of the data for factor analysis was assessed using the Kaiser–Meyer–Olkin (KMO) measure of sampling adequacy and Bartlett’s test of sphericity. The KMO statistic evaluates whether items share sufficient common variance to support factor analysis, whereas Bartlett’s test examines whether the inter-item correlations are sufficiently strong to justify this analysis [24].

Factor retention was determined by parallel analysis, which compares initial eigenvalues with criterion values generated from random simulated data of equivalent size, thereby reducing the risk of over-extraction [25]. A factor was retained only when the initial eigenvalue exceeded the corresponding parallel-analysis criterion value. Items were considered to load adequately if their factor loading was 0.40 or greater. Initial eigenvalues, parallel-analysis criterion values, factor loadings, and variance explained were reported. For the initial two-factor solution, factor loadings were rotated using Oblimin with Kaiser normalization [26].

Internal consistency was assessed using Cronbach’s alpha (α) and McDonald’s omega (ω) [27,28]. Cronbach’s alpha examined the degree of inter-relatedness among scale items, whereas McDonald’s omega additionally considered differences in item loadings on the underlying factor [28,29]. For the five newly added items, item-level performance was further evaluated using means, standard deviations, corrected item–rest correlations, Cronbach’s alpha if the item was deleted, and one-factor loadings to assess their contribution to the internal consistency and structural validity of the extended aVHS [29].

#### 2.5.3. Evaluation of Criterion Validity

Criterion validity was examined using receiver operating characteristic (ROC) analysis, with the qualitative focus-group classification of VH as the reference standard. The area under the curve (AUC), with 95% confidence intervals, was used to assess how well each scale distinguished between hesitant (very and somewhat hesitant) and non-hesitant participants, as classified by the qualitative analysis of the focus-group data [30]. Classification performance metrics for each scale were then derived from logistic regression predicted probabilities using a threshold of ≥0.50. Participants with a predicted probability ≥0.50 were classified as vaccine-hesitant [31]. Sensitivity represented the proportion of vaccine-hesitant individuals correctly identified by each scale, whereas specificity represented the proportion of non-hesitant individuals correctly identified. Positive predictive value (PPV) reflected the proportion of scale-classified hesitant participants who were also classified as hesitant by the focus-group reference standard, while negative predictive value (NPV) reflected the corresponding proportion among scale-classified non-hesitant participants. Overall accuracy reflected the total proportion of participants correctly classified as hesitant or non-hesitant. These metrics were used to assess psychometric classification performance, not to define clinical diagnostic cutoffs [31,32].

As a sensitivity analysis, multivariable linear regression models were used with standardized original and extended aVHS scores as outcomes. These models examined whether country of residence was independently associated with standardized vaccine hesitancy scores after adjustment for age, gender, and educational attainment. All analyses were performed using Stata version 14.2.

### 2.6. Ethical Considerations

The study was conducted in accordance with the Declaration of Helsinki and was approved by the Cyprus National Bioethics Committee. Participation was voluntary, and all participants provided informed consent before completing the questionnaire.

## 3. Results

### 3.1. Participant Characteristics

The study sample comprised 491 participants, while the focus-group sample consisted of 68 participants (Table 1). The majority of the study sample’s participants were women (64.2%), while nearly half of the focus-group participants were men (45.6%), In both samples, the most common age groups were 31–40 years (28.3% vs. 29.4%) and 41–50 years (29.3% vs. 32.3%) for the study sample and focus groups, respectively. Based on the focus-group classification, participants were most commonly classified as either not hesitant or somewhat hesitant (39.7% each), while 20.6% were classified as very hesitant. Finally, the focus-group sample showed higher mean vaccine hesitancy scores than the study sample on both the original aVHS (29.3 ± 8.3 vs. 25.0 ± 7.4) and the extended aVHS (43.6 ± 12.8 vs. 38.0 ± 11.1).

### 3.2. Structural Validity and Internal Consistency

Sampling adequacy was excellent for both scales. The Kaiser–Meyer–Olkin value was 0.926 for the original aVHS and 0.953 for the extended aVHS, indicating that both item correlation matrices were highly suitable for factor analysis. Bartlett’s test of sphericity was statistically significant for both scales (original aVHS: χ^2^(45) = 3251.06, *p* < 0.001; extended aVHS: χ^2^(105) = 5733.40, *p* < 0.001), further confirming factorability.

Parallel analysis of the initial eigenvalues supported a one-factor solution for both scales in Greek-speaking populations (Table 2). For the original aVHS, the first initial eigenvalue (6.031) exceeded the corresponding parallel-analysis criterion value (1.226). Similarly, for the extended aVHS, the first initial eigenvalue (9.009) exceeded its criterion value (1.304), whereas the second initial eigenvalue (1.108) did not exceed the corresponding criterion value (1.237), supporting retention of a single factor.

The retained one-factor EFA solution demonstrated strong loadings across all items of both scales (Table 3). Loadings ranged from 0.67 to 0.86 for the original aVHS and from 0.65 to 0.87 for the extended aVHS. The single factor explained a substantial and nearly identical proportion of total item variance in the original and extended aVHS (60.31% and 60.06%, respectively), indicating that the addition of the five new items preserved the common one-factor structure of the scale. Internal consistency was excellent for both versions, and, in fact, our extended aVHS had slightly better metrics: Cronbach’s α = 0.92 and McDonald’s ω = 0.93 for the original aVHS, and Cronbach’s α = 0.95 and McDonald’s ω = 0.95 for the extended aVHS. A single-factor solution was also supported by the original psychometric evaluation of the Greek version of the aVHS (Cronbach’s α = 0.884) [16].

As a supplementary EFA, an initial two-factor solution for the extended aVHS was examined to assess whether risk-perception items formed a distinct dimension (Supplementary Table S4). Some risk-related items loaded more strongly on the second factor; however, the second initial eigenvalue (1.108) did not exceed the corresponding parallel-analysis criterion value (1.237). Therefore, the one-factor solution was retained as the final structure.

Item-level psychometric properties of the five newly added items were also evaluated (Supplementary Table S5). Corrected item–rest correlations ranged from 0.69 to 0.85, indicating that each added item was correlated with the remainder of the extended scale. Cronbach’s α if the item was deleted ranged from 0.945 to 0.949, suggesting that the removal of any of the five added items would not improve the internal consistency. One-factor loadings ranged from 0.72 to 0.87, supporting the contribution of the added items to the common vaccine hesitancy factor and indicating that the new risk- and safety-related items were psychometrically integrated within the extended scale.

Based on the one-factor solution noted above and using the same five-point Likert response format as the original scale, the total extended aVHS score was calculated by summing all 15 items. Possible scores ranged from 15 to 75, with higher scores indicating greater vaccine hesitancy and lower scores indicating lower vaccine hesitancy. The English and Greek wording and scoring are presented in Supplementary Tables S2 and S3.

### 3.3. Criterion Validity Against Focus-Group Classification

**Figure 1:**
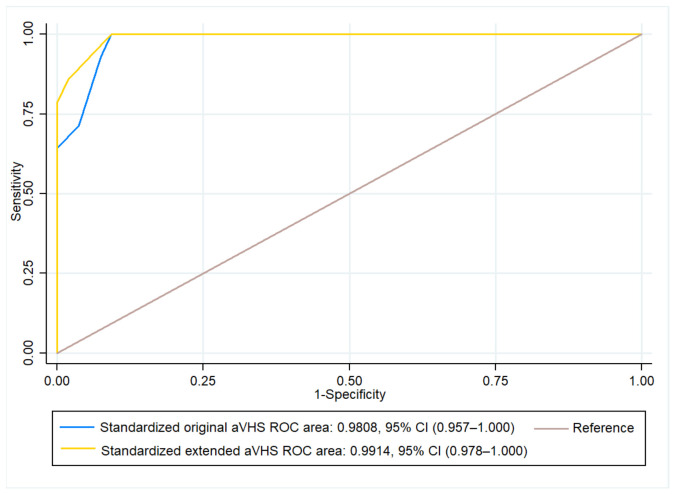
Receiver operating characteristic (ROC) curves for the standardized original and extended adult Vaccine Hesitancy Scale (aVHS) for classifying generally vaccine-hesitant participants.

Both the original and extended aVHS demonstrated excellent criterion validity against focus-group-defined general vaccine hesitancy. ROC analysis using standardized scale scores showed near-perfect discriminatory ability for both versions, with a slightly higher AUC for the extended aVHS than for the original aVHS (0.991, 95% CI: 0.978–1.000 vs. 0.981, 95% CI: 0.958–1.000) (Figure 1). The lower bounds of the 95% confidence intervals were also within the range of excellent discriminatory performance, supporting the robustness of the AUC estimates across the combined study population.

Based on the logistic regression classification results, the extended aVHS showed numerically better performance than the original scale across all classification indices (Table 4). Compared with the original aVHS, the extended aVHS showed higher sensitivity (85.7% vs. 71.4%) while maintaining very high specificity (98.2% vs. 96.3%). This indicates that the extended scale identified a slightly higher proportion of participants classified as vaccine-hesitant by the qualitative reference standard, while still correctly classifying a very high proportion of non-hesitant participants.

The raw classification counts (Supplementary Table S6) indicated fewer false-positive and false-negative classifications for the extended aVHS. This was reflected in the higher PPV for the extended than the original scale (92.3% vs. 83.3%), indicating that a higher proportion of participants classified as hesitant were correctly identified as hesitant by the focus-group reference standard. The extended aVHS also showed a higher NPV than the original scale (96.4% vs. 92.9%), indicating that a higher proportion of participants classified as non-hesitant were correctly identified as non-hesitant. Overall, these findings indicate that classifications based on the extended scale were more consistently aligned with the focus-group reference classification. Overall accuracy was also higher for the extended aVHS than for the original scale (95.6% vs. 91.2%), indicating that the extended scale correctly classified a greater total proportion of participants as either hesitant or non-hesitant.

Univariate linear regression analysis for confirmation indicated that, in the focus-group sample, for both scales, participants classified as not hesitant had the lowest mean scores, followed by those classified as somewhat hesitant, while the highest scores were recorded among those classified as very hesitant. This pattern appeared more pronounced for the extended aVHS (31.5 ± 7.2, 46.8 ± 6.4, and 60.8 ± 4.4, respectively; *p* < 0.001) than for the original aVHS (21.4 ± 4.7, 31.4 ± 4.2, and 40.3 ± 3.0, respectively; *p* < 0.001).

Finally, sensitivity analysis using standardized scale scores showed that country of residence was not independently associated with either the original or extended aVHS score after adjustment for age, gender, and educational attainment (original aVHS: Cyprus vs. Greece, β = 0.163, 95% CI: −0.012 to 0.339, *p* = 0.068; extended aVHS: β = 0.119, 95% CI: −0.056 to 0.295, *p* = 0.182).

### 3.4. Distribution of Original and Extended aVHS Scores Across Participant Characteristics

Original and extended aVHS scores showed broadly similar patterns across participant characteristics (Table 5). Scores on both scales did not differ significantly by sex, country or area of residence, or marital status (*p* > 0.05). In contrast, scores varied significantly by age. Mean scores were highest among participants aged 18–30 years and lowest among those aged 31–40 years for both the original aVHS (27.1 ± 8.0 vs. 23.5 ± 6.5, *p* = 0.005) and the extended aVHS (41.3 ± 11.8 vs. 35.8 ± 9.8, *p* = 0.004). The remaining age groups showed relatively close mean scores, especially for the original scale. Scores also decreased significantly with increasing educational attainment, with the highest mean scores recorded among participants with up to high school education and the lowest among those with a postgraduate (MSc or PhD) university education for both the original aVHS (28.3 ± 7.5 vs. 21.7 ± 6.6, *p* < 0.001) and the extended aVHS (43.0 ± 11.1 vs. 33.0 ± 10.1, *p* < 0.001). A similar gradient was observed for monthly income, with the highest scores among participants with income ≤€1000 and the lowest among those with income >€2000 for both the original aVHS (27.3 ± 7.5 vs. 22.4 ± 7.5, *p* < 0.001) and the extended aVHS (41.6 ± 11.0 vs. 34.0 ± 11.4, *p* < 0.001).

## 4. Discussion

This study developed and psychometrically evaluated an extended version of the adult Vaccine Hesitancy Scale (aVHS) among Greek-speaking adults in Greece and Cyprus. The extended scale retained a coherent one-factor structure, showed excellent internal consistency, strong item-level performance for the newly added items, and excellent criterion validity against a carefully derived qualitative focus-group classification. These findings support the extended aVHS as a broader, psychometrically coherent improved version of the original aVHS.

More specifically, this study strengthens the enhancement of the original scale with additional risk- and safety-related vaccine concern items. To our knowledge, this is the first successful attempt to extend the aVHS in response to the gaps noted in previous parental and adult VHS validation work [11,13,15,16]. Compared to the previous Greek validation of the original 10-item aVHS [16], this study evaluated a 15-item version with broader vaccine risk and safety content and tested its criterion validity against focus-group classification. Thus, the scale was assessed not only for structural validity and internal consistency, but also for its ability to identify individuals with vaccine-hesitant attitudes.

These added items capture domains central to adult vaccine decision-making, especially during the introduction of new vaccines or periods of uncertainty. These include long-term safety concerns, risk–benefit appraisal, perceived probability of serious vaccine-related harm, scientific credibility and safety of vaccination, as well as perceived alternatives to vaccination [5,6,9,10]. Although highlighted during the COVID-19 pandemic, these concerns extend across broader vaccination contexts. Thus, the extended scale assesses risk- and safety-related concerns with greater conceptual depth than the original aVHS.

Given the conceptual depth of the added risk- and safety-related items, cross-cultural adaptation emphasized conceptual and contextual equivalence rather than literal translation. This was particularly important for reverse-scored and risk-perception items that are sensitive to wording nuances. Participant paraphrasing supported the comprehensibility and face validity of the Greek wording, strengthening the suitability of the extended aVHS for assessing vaccine hesitancy among Greek-speaking adults in Greece and Cyprus.

With respect to scale structure, previous VHS validation studies often reported two-factor solutions, generally reflecting confidence- and risk-related dimensions [11,13,15,16]. In the present study, however, parallel analysis supported a single factor for both the original and extended aVHS. This suggests that vaccine confidence, risk perception, and safety-related concerns functioned as closely connected components of a common VH construct in this Greek-speaking adult sample. Because the five added items represented newly developed content with no prior empirical structure, EFA was employed in this first development phase, while parallel analysis provided a rigorous criterion for factor retention, helping avoid over-interpretation of dimensions not supported by the data [20,21,25].

Importantly, the absence of a retained second factor does not weaken the value of the extended scale. Rather, it supports a clear one-factor structure in which the added risk- and safety-related items broadened content coverage while remaining psychometrically integrated within the common vaccine hesitancy factor. The added items showed adequate one-factor loadings and corrected item–rest correlations, and deleting any of them would not have improved internal consistency. Thus, the extended aVHS strengthened the assessment of risk- and safety-related concerns without increasing structural complexity or weakening psychometric coherence.

Consistent with this structural analysis, the explained variance findings further support the one-factor interpretation. The one-factor solution accounted for a substantial and nearly identical proportion of total item variance in both scale versions, indicating that the five newly added items did not dilute the common latent structure. This is a practical advantage for population-based research, as a single total score can facilitate the monitoring of vaccine hesitancy levels, identification of higher-risk groups, and planning of targeted vaccination-related public health interventions [10,11].

In line with the structural findings, the reliability results further support the coherence of the extended aVHS. The agreement between Cronbach’s alpha and McDonald’s omega provides convergent evidence for its excellent reliability. Importantly, the extended aVHS showed slightly higher reliability than the original scale despite incorporating new risk- and safety-related items. This indicates that the added items contributed coherently to the common VH construct, rather than introducing measurement noise or weakening the scale’s internal consistency.

The criterion validity findings further support the added value of the extended aVHS. Using attitudes expressed in the focus groups as the reference standard was a methodological strength because VH is fundamentally attitudinal and cannot be fully captured by vaccination status alone [7,8,10]. The focus-group questions explored vaccine safety, long-term concerns, scientific and institutional trust, development speed and rigor, risk–benefit appraisal, health system reliability, and perceived alternatives to vaccination. These domains were closely aligned with the extended scale and addressed the limited risk- and safety-related coverage previously noted in parental and adult VHS validation work [11,13,15,16]. Although both scale versions showed excellent discriminatory ability, the extended aVHS had a more balanced classification profile, with an improved identification of hesitant participants while maintaining a very high correct classification of non-hesitant participants. This supports its practical value for identifying adults with vaccine-hesitant attitudes and informing targeted public health interventions before hesitancy is reflected in vaccine delay or refusal. Importantly, this added value was achieved with only five additional items, suggesting broader risk- and safety-related assessment with limited increase in respondent burden.

Sociodemographic score patterns further support the interpretability and practical relevance of the extended aVHS. Higher scores among younger adults and participants with lower educational attainment and income were consistent with international evidence linking VH to perceived disease risk, socioeconomic resources, access to trustworthy information, and confidence in institutions [7,8,9,10,17]. The presence of these gradients across both scale versions supports the construct validity of the extended aVHS and indicates that the added items did not alter expected sociodemographic patterns of VH. By contrast, scores did not differ significantly by sex, country of residence, area of residence, or marital status, suggesting that socioeconomic factors were more consistently associated with VH in this sample than broader demographic or geographic characteristics. The adjusted sensitivity analysis further strengthened the combined Greece–Cyprus evaluation, indicating that country of residence did not primarily explain the observed score patterns after accounting for key demographic differences. This supports the use of the extended aVHS across these two Greek-speaking populations.

The specific study has several limitations which are acknowledged here. Random probability sampling was not feasible during and immediately after the COVID-19 pandemic; however, the targeted quota-sampling approach achieved demographic, geographic, and socioeconomic heterogeneity at a satisfactory extent and enabled recruitment across both Greece and Cyprus under restrictive fieldwork conditions. The focus-group subsample included a higher proportion of Cyprus residents than the main validation sample, which should be considered when interpreting the criterion validity findings. Country-stratified ROC analyses were not performed because the small number of Greece-based participants in the focus-group subsample would have produced statistically unstable estimates. However, country of residence was not independently associated with standardized original or extended aVHS scores in sensitivity analyses adjusted for age, gender, and educational attainment. Therefore, potential country-specific influences on the qualitative reference classification cannot be fully excluded, and replication in larger, geographically balanced validation samples is warranted.

Furthermore, item 15 combined perceived safety and ease/convenience of alternatives to vaccination within a single statement, which may introduce some conceptual ambiguity. Although the item performed adequately in the present psychometric evaluation, future studies could examine whether separate items for perceived safety and perceived ease of alternatives provide greater conceptual precision.

Finally, although this study provides the first psychometric evaluation of the extended aVHS, further evaluation in the English language, as well as in other languages or cultural settings, is needed to examine its stability, applicability, and wider international use.

## 5. Conclusions

The newly developed extended aVHS provides a more comprehensive assessment of adult VH, retaining the strengths of the original 10-item scale while improving coverage of risk- and safety-related concerns. It approaches VH as an attitudinal public health construct, rather than as a clinical diagnosis by capturing long-term safety concerns, risk–benefit appraisal, scientific credibility, and perceived alternatives with greater conceptual depth. This broader assessment may help identify both VH and the concerns that shape it, supporting more targeted vaccine hesitancy surveillance, identification of groups with higher hesitancy, and the design of appropriate vaccination communication strategies. Its contribution is particularly relevant when new vaccines are introduced or when uncertainty about vaccine safety and trust influences adult vaccination decisions.

## Figures and Tables

**Table 1:** Characteristics of the study participants and focus-group subsample.

	Study Participants (n = 491)	Focus Groups (n = 68)
**Gender [n (%)]**		
Men	176 (35.8)	31 (45.6)
Women	315 (64.2)	37 (54.4)
**Age [n (%)]**		
18–30	100 (20.4)	11 (16.2)
31–40	139 (28.3)	20 (29.4)
41–50	144 (29.3)	22 (32.3)
51–60	46 (9.4)	4 (5.9)
>60	62 (12.6)	11 (16.2)
**Country of residence [n (%)]**		
Greece	291 (59.3)	13 (19.1)
Cyprus	200 (40.7)	55 (80.9)
**Area of residence [n (%)]**		
Urban	418 (85.1)	53 (77.6)
Semi-urban/rural	73 (14.9)	15 (22.4)
**Marital status [n (%)] ^a^**		
Single	155 (31.7)	23 (33.8)
Married/cohabiting	294 (60.1)	39 (57.4)
Divorced/separated/widowed	40 (8.2)	6 (8.8)
**Educational attainment [n (%)]**		
Up to high school	114 (23.2)	22 (32.4)
College/BSc	222 (45.2)	30 (44.1)
MSc/PhD	155 (31.6)	16 (23.5)
**Income [n (%)] ^b^**		
≤1000	174 (36.9)	15 (22.1)
1001–2000	188 (39.9)	27 (39.7)
>2000	109 (23.1)	26 (38.2)
**General vaccine hesitancy** **(based on focus groups) [n (%)]**		
Not hesitant	-	27 (39.7)
Somewhat hesitant	-	27 (39.7)
Very hesitant	-	14 (20.6)
**Original aVHS score [mean score (SD)]**	25.0 (7.4)	29.3 (8.3)
**Extended aVHS score [mean score (SD)]**	38.0 (11.1)	43.6 (12.8)

aVHS, adult Vaccine Hesitancy Scale; -, not applicable; ^a^. n = 489, ^b^. n = 471. Note: The focus-group subsample included a higher proportion of Cyprus residents because the qualitative focus-group phase required in-person participation and was more constrained by logistical feasibility. This geographic imbalance is acknowledged in the Limitations Section and was considered when interpreting the criterion validity findings.

**Table 2:** Initial EFA eigenvalues and parallel-analysis criterion values for the original and extended adult Vaccine Hesitancy Scale (aVHS).

	Original aVHS (10 Items)		Extended aVHS (15 Items)	
	EFA	Parallel Analysis	EFA	Parallel Analysis
Factor 1	6.031	1.226	9.009	1.304
Factor 2	-	-	1.108	1.237

**Table 3:** One-factor EFA loadings and internal consistency of the original and extended adult Vaccine Hesitancy Scale (aVHS).

	Original aVHS (10 Items)	Extended aVHS (15 Items)
**Original aVHS Items**		
1. Vaccines are important for my health.	0.84	0.82
2. Vaccines are effective.	0.80	0.79
3. Being vaccinated is important for the health of others in my country.	0.81	0.78
4. All vaccines offered by the government in my country are beneficial.	0.86	0.83
5. New vaccines carry more risks than older vaccines.	0.71	0.73
6. The information I receive about vaccines offered in my country is reliable and trustworthy.	0.78	0.76
7. Getting vaccines is a good way to protect myself from disease.	0.84	0.83
8. Generally I do what my doctor or health care provider recommends about vaccines.	0.73	0.71
9. I am concerned about serious adverse effects of vaccines.	0.70	0.72
10. I do not need vaccines for diseases that are not common anymore.	0.67	0.65
**Newly added aVHS Items**		
11. I worry that the side effects of vaccines may not be seen immediately but in the long term (i.e., in the future).	-	0.72
12. The risks from vaccines are greater than the protection they offer.	-	0.87
13. The possibility of something serious happening to me as a result of vaccination is extremely small.	-	0.79
14. Vaccination is a scientifically proven and safe way of health protection.	-	0.84
15. There are much safer and easier ways than vaccination for protecting against communicable diseases.	-	0.74
% Variance	60.31	60.06
**Cronbach’s α**	**0.92**	**0.95**
**McDonald’s ω**	**0.93**	**0.95**

Note: Items 11–15 are newly added risk- and safety-related items developed to broaden the original aVHS in line with the WHO SAGE 3Cs framework and previous recommendations to strengthen risk-perception coverage. They assess long-term safety concerns, risk–benefit appraisal, perceived vaccine-related harm, scientific credibility/safety, and perceived alternatives to vaccination.

**Table 4:** Criterion validity performance of the original and extended adult Vaccine Hesitancy Scale (aVHS) against focus-group classification.

	Original aVHS Performance Based on Focus-Group Status ^a^	Extended aVHS Performance Based on Focus-Group Status ^a^
AUC (95% CI)	0.981 (0.958–1.000)	0.991 (0.978–1.000)
Sensitivity (%)	71.4	85.7
Specificity (%)	96.3	98.2
PPV (%)	83.3	92.3
NPV (%)	92.9	96.4
Accuracy (%)	91.2	95.6

AUC, area under the curve; 95% CI, 95% confidence interval; PPV, positive predictive value; NPV, negative predictive value. ^a^ General vaccine hesitancy as determined by qualitative focus-group evaluation. Classification by the original and extended aVHS was based on logistic regression against the focus-group classification; participants with predicted probability ≥0.5 were classified as vaccine-hesitant.

**Table 5:** Original and extended adult Vaccine Hesitancy Scale (aVHS) scores by participant characteristics.

	Original aVHS Score [Mean Score (SD)]	Extended aVHS Score [Mean Score (SD)]
**Gender**		
Men	25.5 (8.1)	38.4 (11.9)
Women	24.8 (7.0)	37.8 (10.6)
*p*-value	0.309	0.565
**Age-group**		
18–30	27.1 (8.0)	41.3 (11.8)
31–40	23.5 (6.5)	35.8 (9.8)
41–50	24.9 (8.0)	37.8 (12.0)
51–60	25.9 (7.8)	39.3 (11.5)
>60	24.5 (5.8)	37.0 (8.9)
*p*-value	0.005	0.004
**Country of residence**		
Greece	24.6 (7.2)	37.6 (10.7)
Cyprus	25.6 (7.7)	38.6 (11.6)
*p*-value	0.159	0.344
**Area of residence**		
Urban	25.0 (7.3)	37.9 (11.0)
Semi-urban/rural	26.0 (7.9)	39.4 (11.8)
*p*-value	0.256	0.258
**Marital status**		
Single	25.6 (7.4)	38.7 (11.0)
Married/cohabiting	24.5 (7.5)	37.4 (11.2)
Divorced/separated/widowed	26.4 (6.9)	39.6 (10.1)
*p*-value	0.166	0.314
**Educational attainment**		
Up to high school	28.3 (7.5)	43.0 (11.1)
College/BSc	25.7 (7.0)	38.9 (10.3)
MSc/PhD	21.7 (6.6)	33.0 (10.1)
*p*-value	<0.001	<0.001
**Income**		
≤1000	27.3 (7.5)	41.6 (11.0)
1001–2000	24.6 (6.9)	37.3 (10.3)
>2000	22.4 (7.5)	34.0 (11.4)
*p*-value	<0.001	<0.001
**General vaccine hesitancy** (Based on focus groups)		
Not hesitant	21.4 (4.7)	31.5 (7.2)
Somewhat hesitant	31.4 (4.2)	46.8 (6.4)
Very hesitant	40.3 (3.0)	60.8 (4.4)
*p*-value	<0.001	<0.001

Note: Values are mean scores with standard deviation (SD). Higher scores indicate greater vaccine hesitancy.

## Data Availability

The data presented in this study are available on request from the corresponding authors. The raw data used in the present study include sensitive anonymous participant information and are bound by local Bioethics regulations, and therefore cannot be distributed or made publicly available.

## Supplementary Materials

The following supporting information can be downloaded at: https://www.mdpi.com/article/10.3390/vaccines14070628/s1, Supplementary Table S1: Items and scoring of the original adult Vaccine Hesitancy Scale (aVHS); Supplementary Table S2: Items and scoring of the extended adult Vaccine Hesitancy Scale (aVHS), English version; Supplementary Table S3: Items and scoring of the extended adult Vaccine Hesitancy Scale (aVHS), Greek version; Supplementary Table S4: Initial exploratory two-factor EFA solution examined for the extended aVHS; Supplementary Table S5: Item-total correlations, internal consistency, and one-factor loadings for the five newly added items of the extended aVHS; Supplementary Table S6: Classification of participants as generally vaccine-hesitant based on the original and extended aVHS.
