# Supplementary material for: Development and Validation of an Extended Adult Vaccine Hesitancy Scale in Greek-Speaking Populations
Source: Vaccines (Basel). 2026 Jul 17;14(7):628. doi: 10.3390/vaccines14070628 (PMC13418891; doi:10.3390/vaccines14070628)
Supplement: Supplementary file 1 [file vaccines-14-00628-s001.zip › vaccines-4400999-supplementary.pdf]

Supplementary material

Supplementary Table S1. Items and scoring of the original adult vaccine hesitancy scale (aVHS) in English.

	Strongly disagree	Disagree	Neither agree nor disagree	Agree	Strongly agree
1. Vaccines are important for my health.	5	4	3	2	1
2. Vaccines are effective.	5	4	3	2	1
3. Being vaccinated is important for the health of others in my community.	5	4	3	2	1
4. All routine vaccination recommended by the government programme in my community are beneficial.	5	4	3	2	1
5. New vaccines carry more risks than older vaccines.	1	2	3	4	5
6. The information I receive about vaccines from the vaccine program is reliable and trustworthy.	5	4	3	2	1
7. Getting vaccines is a good way to protect myself from disease.	5	4	3	2	1
8. Generally I do what my doctor or healthcare provider recommends about vaccines.	5	4	3	2	1
9. I am concerned about serious adverse effects of vaccines.	1	2	3	4	5
10. I do not need vaccines for diseases that are not common anymore.	1	2	3	4	5

Note: The initial prompt is **"***How much do you agree with each of the following statements on vaccinations?"*

Supplementary Table S2. Items and scoring of the extended adult vaccine hesitancy scale (aVHS), in English.

	Strongly disagree	Disagree	Neither agree nor disagree	Agree	Strongly agree
1. Vaccines are important for my health.	5	4	3	2	1
2. Vaccines are effective.	5	4	3	2	1
3. Being vaccinated is important for the health of others in my country.	5	4	3	2	1
4. All vaccines offered by the government in my country are beneficial.	5	4	3	2	1
5. New vaccines carry more risks than older vaccines.	1	2	3	4	5
6. The information I receive about vaccines offered in my country is reliable and trustworthy.	5	4	3	2	1
7. Getting vaccines is a good way to protect myself from disease.	5	4	3	2	1
8. Generally I do what my doctor or health care provider recommends about vaccines.	5	4	3	2	1
9. I am concerned about serious adverse effects of vaccines.	1	2	3	4	5
10. I do not need vaccines for diseases that are not common anymore.	1	2	3	4	5
11. I worry that the side effects of vaccines may not be seen immediately but in the long term (i.e. in the future).	1	2	3	4	5
12. The risks from vaccines are greater than the protection they offer.	1	2	3	4	5
13. The possibility of something serious happening to me as a result of vaccination is extremely small.	5	4	3	2	1
14. Vaccination is a scientifically proven and safe way of health protection.	5	4	3	2	1
15. There are much safer and easier ways than the vaccination for protection against communicable diseases.	1	2	3	4	5

Note: The initial prompt is **“***How much do you agree with each of the following statements on vaccinations?”*

Supplementary Table S3. Items and scoring of the extended adult vaccine hesitancy scale (aVHS), in Greek.

	Διαφωνώ απόλυτα	Διαφωνώ	Ούτε συμφωνώ, ούτε διαφωνώ	Συμφωνώ	Συμφωνώ απόλυτα
1. Τα εμβόλια είναι σημαντικά για την υγεία μου.	5	4	3	2	1
2. Τα εμβόλια είναι αποτελεσματικά.	5	4	3	2	1
3. Το να εμβολιαστώ είναι σημαντικό για την υγεία των άλλων ανθρώπων στη χώρα μου.	5	4	3	2	1
4. Όλα τα εμβόλια που προσφέρει η κυβέρνηση στη χώρα μου είναι ωφέλιμα.	5	4	3	2	1
5. Τα νέα εμβόλια ενέχουν περισσότερους κινδύνους από τα παλαιότερα εμβόλια.	1	2	3	4	5
6. Οι πληροφορίες που λαμβάνω σχετικά με τα εμβόλια που προσφέρονται στη χώρα μου είναι αξιόπιστες και μπορώ να τις εμπιστευτώ.	5	4	3	2	1
7. Η λήψη εμβολίων είναι ένας καλός τρόπος για να προστατευτώ από ασθένειες.	5	4	3	2	1
8. Γενικά κάνω ό,τι συνιστά ο γιατρός μου (ή κάποιος άλλος υπεύθυνος επαγγελματίας υγείας) σχετικά με τα εμβόλια.	5	4	3	2	1
9. Ανησυχώ για σοβαρές παρενέργειες των εμβολίων.	1	2	3	4	5
10. Δεν χρειάζομαι εμβόλια για ασθένειες που δεν είναι πια συχνές.	1	2	3	4	5
11. Ανησυχώ ότι οι παρενέργειες των εμβολίων μπορεί να μη φαίνονται άμεσα αλλά μακροπρόθεσμα (δηλαδή στο μέλλον).	1	2	3	4	5
12. Οι κίνδυνοι από τα εμβόλια είναι μεγαλύτεροι από την προστασία που μπορεί να προσφέρουν.	1	2	3	4	5
13. Η πιθανότητα να πάθω κάτι σοβαρό εξαιτίας του εμβολιασμού είναι εξαιρετικά μικρή.	5	4	3	2	1
14. Ο εμβολιασμός είναι ένας επιστημονικά κατοχυρωμένος και ασφαλής τρόπος προστασίας της υγείας.	5	4	3	2	1
15. Υπάρχουν πολύ πιο ασφαλείς και εύκολοι τρόποι από τον εμβολιασμό, για προστασία από μεταδοτικά νοσήματα.	1	2	3	4	5

Note: The initial prompt is “*Σας παρακαλούμε να δηλώσετε κατά πόσον συμφωνείτε με τις ακόλουθες δηλώσεις σχετικά με τον εμβολιασμό γενικότερα (δεν αφορούν συγκεκριμένα τη νόσο COVID-19).*”

Supplementary Table S4. Initial exploratory two-factor EFA solution examined for the extended adult vaccine hesitancy scale (aVHS).

	Extended aVHS (15 items)	
Items	Factor 1	Factor 2
1. Vaccines are important for my health.	0.90	
2. Vaccines are effective.	0.94	
3. Being vaccinated is important for the health of others in my country.	0.90	
4. All vaccines offered by the government in my country are beneficial.	0.71	
5. New vaccines carry more risks than older vaccines.		0.649
6. The information I receive about vaccines offered in my country is reliable and trustworthy.	0.47	
7. Getting vaccines is a good way to protect myself from disease.	0.92	
8. Generally I do what my doctor or health care provider recommends about vaccines.	0.66	
9. I am concerned about serious adverse effects of vaccines.		0.895
10. I do not need vaccines for diseases that are not common anymore.	0.44	
11. I worry that the side effects of vaccines may not be seen immediately but in the long term (i.e. in the future).		0.933
12. The risks from vaccines are greater than the protection they offer.[a]	0.53	0.438
13. The possibility of something serious happening to me as a result of vaccination is extremely small.	0.54	
14. Vaccination is a scientifically proven and safe way of health protection.	0.83	
15. There are much safer and easier ways than vaccination for protecting against communicable diseases.	0.56	
% variance	60.06	7.39
Cronbach's α	0.94	0.85

Note: Factor loadings were rotated using Oblimin with Kaiser normalization. This initial two-factor solution was examined exploratorily, but the second factor was not retained because parallel analysis supported a one-factor structure.

[a] The item was included in factor 1, because its loading was higher on this factor.

Supplementary Table S5. Item-total correlations, internal consistency, and one-factor loadings for the five added items of the extended adult vaccine hesitancy scale (aVHS).

Added item	Mean	Standard deviation (SD)	Corrected item-rest correlation	Cronbach's α if item deleted	One-factor loading
11. I worry that the side effects of vaccines may not be seen immediately but in the long term (i.e. in the future).	3.49	1.03	0.69	0.949	0.72
12. The risks from vaccines are greater than the protection they offer.	2.40	1.04	0.85	0.945	0.87
13. The possibility of something serious happening to me as a result of vaccination is extremely small.	2.54	0.96	0.75	0.948	0.79
14. Vaccination is a scientifically proven and safe way of health protection.	2.08	0.85	0.80	0.947	0.84
15. There are much safer and easier ways than vaccination for protecting against communicable diseases.	2.45	0.88	0.70	0.949	0.74

Note: One-factor loadings correspond to the final retained one-factor solution of the extended aVHS.

Supplementary Table S6. Classification of participants as generally vaccine hesitant based on the original and extended adult vaccine hesitancy scale (aVHS).

Scale-based classification	General vaccine hesitancy according to focus group[a]		
	Vaccine hesitant	Non-vaccine hesitant	Total
Original aVHS			
Vaccine hesitant	10	2	12
Non-vaccine hesitant	4	52	56
Total	14	54	68
Extended aVHS			
Vaccine hesitant	12	1	13
Non-vaccine hesitant	2	53	55
Total	14	54	68

[a] General vaccine hesitancy as determined by qualitative focus-group evaluation.

Note: Classification by the original and extended aVHS was based on logistic regression against the focus-group classification; participants with predicted probability ≥0.50 were classified as vaccine hesitant. Classification performance metrics derived from these counts are presented in Table 4 of the main manuscript.
